# Correspondence about the article: Asthma in the Brazilian Unified Health Care System: an epidemiological analysis from 2008 to 2021

**DOI:** 10.36416/1806-3756/e20240196

**Published:** 2024-09-16

**Authors:** Marcelo Fouad Rabahi, Amanda da Rocha Oliveira Cardoso, José Eduardo Delfini Cançado

**Affiliations:** 1. Departamento de Pneumologia, Faculdade de Medicina, Universidade Federal de Goiás, Goiânia (GO) Brasil.; 2. Departamento de Pneumologia, Hospital das Clínicas da Universidade Federal de Goiás/Ebserh, Universidade Federal de Goiás, Goiânia (GO) Brasil, Bolsista da FAPEG, n 202310267000719.; 3. Departamento de Pneumologia, Faculdade de Ciências Médicas da Santa Casa de São Paulo, São Paulo (SP) Brasil.

We received with great interest the article published in the *Jornal Brasileiro de Pneumologia*, authored by Pinheiro et al.^1^ and titled “Asthma in the Brazilian Unified Health Care System: an epidemiological analysis from 2008 to 2021”. Their article contains important data on mortality and hospitalizations associated with asthma in Brazil between 2008 and 2021 in the Brazilian *Sistema Único de Saúde* (SUS, Unified Health Care System). The data were extracted from the Information Technology Department of the SUS (DATASUS) in 2022. The authors concluded that the number of deaths and hospitalizations for asthma decreased over the course of the period studied.

Our group analyzed asthma mortality data (ICD-10 codes J45 and J46) in Brazil from the National *Sistema de Informação sobre Mortalidade* (SIM, Mortality Database),^2^ and observed that the number of asthma-related deaths is 3 to 6 times higher than that obtained by the DATASUS.^3^ Pinheiro et al.^1^ found that there were more than 8,000 asthma-related deaths in the 2008-2021 period. In the same period, according to SIM data, there were 34,163 deaths attributed to asthma: 2,696 in 2008 and 2,802 in 2022. Therefore, in 2022, there were an alarming seven asthma-related deaths per day in Brazil,^3^ which is in contrast with the one death per day reported by Pinheiro et al.^1^

Given this context, it is important to emphasize that the mortality data generated by DATASUS come from the outcome “in-hospital death” from the “paid during the period” authorized hospital admissions (AHAs). This information reflects asthma-related deaths occurring only during hospitalization for the disease. On the other hand, the SIM provides information derived from death certificates and thus reports deaths occurring in the public and private health care systems, as well as those occurring outside the hospital setting. Consequently, if the AHA had been generated for a condition other than asthma, an in-hospital death attributed to asthma on the death certificate would not have been counted as an asthma-related death by the DATASUS, which constitutes an additional factor that differentiates DATASUS data from SIM data.

We stress that asthma-related mortality in Brazil has increased over the last 10 years, and that the mortality rate cited by Pinheiro et al.^1^ reflects data from hospitalizations only within the SUS. We also emphasize that 19% and 67% of the asthma-related deaths in Brazil occurred in adults (≥ 40 and < 60 years of age) and the elderly (≥ 60 years of age), respectively. In conclusion, despite the availability of free asthma medication throughout the country, asthma mortality has been increasing, especially among adults and the elderly. Therefore, it is essential to improve access to the diagnosis and treatment of this disease, which is the second most prevalent respiratory disease in Brazil.

Financial support: AROC is the recipient of a grant from the *Fundação de Amparo à Pesquisa do Estado de Goiás* (FAPEG, Foundation for the Support of Research in the State of Goiás; Grant no. 202310267000719).
