# Supplementary material for: Correspondence about the article: Asthma in the Brazilian Unified Health Care System: an epidemiological analysis from 2008 to 2021
Source: J Bras Pneumol. 2024 Sep 16;50(4):e20240196. doi: 10.36416/1806-3756/e20240196 (PMC11449602; doi:10.36416/1806-3756/e20240196)
Supplement: Related texts

# Authors’ reply

**Authors:** David Halen Araújo Pinheiro, João Victor Hermógenes de Souza, Alberto Fernando Oliveira Justo, Regina Maria Carvalho-Pinto, Fabiano Francisco de Lima, Celso R F Carvalho

**Affiliations:** 1. Departamento de Fisioterapia, Faculdade de Medicina, Universidade de São Paulo - USP - São Paulo (SP) Brasil.; 2. Laboratório de Fisiopatologia do Envelhecimento, Departamento de Clínica Médica, Universidade de São Paulo - USP - São Paulo (SP) Brasil.; 3. Divisão de Pneumologia, Instituto do Coração - InCor - Hospital das Clinicas Faculdade de Medicina, Universidade de São Paulo, São Paulo (SP) Brasil.

We received with great interest the correspondence JBPNEU-2024-0196, containing the comments from Rabahi et al. about our study entitled “Asthma in the Brazilian Unified Health Care System: an epidemiological analysis from 2008 to 2021.”^1^ They suggested that our results demonstrating a reduction in in-hospital asthma-related mortality run contrary to their results showing an increase in such mortality in the last ten years.^2^ We consider that the results of the two studies are not contradictory but rather complementary and can further understanding of what has happened to individuals with asthma during the last decade.

It is widely known that asthma medication is freely available via the Brazilian *Sistema Único de Saúde* (SUS, Unified Health Care System), improving asthma treatment. In our opinion, the apparent difference between the results of the two studies is a consequence of inadequate treatment despite the available medication. When patients receive proper treatment for exacerbations during hospitalization (for instance), the number of deaths can be reduced.

We also emphasize that the two studies differed in terms of the databases consulted, which could explain the distinct results and misinterpretations. We used data from the Information Technology Department of the SUS (DATASUS), which includes only individuals treated via the public health care system, as was well stated by Rabahi et al. in their correspondence (“this information reflects asthma-related deaths occurring only during hospitalization for the disease” which was stated in our article). In contrast, Rabahi et al. used data from the Brazilian National Mortality Database, which includes individuals treated via the public and private health care systems. In addition, the increase in the number of asthma-related deaths observed by Rabahi et al.^2^ represented a “special increase among adults in the last three years (2020 to 2022)”; that is, during the COVID-19 pandemic. This period presented a marked increase in the number of deaths caused by respiratory symptoms in adults and the elderly, which may have resulted in the cause of death being misrepresented. Furthermore, our data show a linear decline in the number of hospitalizations and deaths in the previous ten years, which continued during the COVID-19 pandemic. Moreover, the Brazilian Thoracic Society website recently published data referring to asthma in Brazil (also collected from DATASUS), indicating that in 2022 there were 83,155 hospitalizations for asthma and 524 deaths from the disease, stating that the “expansion of care and access to medications . . . led to a significant drop in hospitalizations for asthma”, corroborating our results.^3^ Finally, our study shows other relevant and associated findings, including hospitalization rates and costs in the various regions of Brazil.

In conclusion, we agree that not all patients with asthma receive proper medical treatment in Brazil, despite the availability of free asthma medication treatment throughout the country, and that we still have a lot of work to do. We are also anxious to carefully analyze the results presented by Rabahi et al.,^2^ which will undoubtedly add important information on asthma-related deaths in Brazil. It will be important to understand whether the increase in the number of deaths was due to asthma itself or was a consequence of the pandemic. 

## References

[1] Pinheiro DHA, Souza JVH, Justo AFO, Carvalho-Pinto RM, Lima FF, Carvalho CRF (2024). Asthma in the Brazilian Unified Health Care System an epidemiological analysis from 2008 to 2021. J Bras Pneumol.

[2] Rabahi MF, Cardoso ARO, Ferreira ACG, Scabello RT, Fonseca JDAV, Zung S (2024). Higher Asthma Mortality in Elders and Female Subjects in Brazil A 10-year Series [abstract]. Am J Respir Crit Care. Med.

[3] Sociedade Brasileira de Pneumologia e Tisiologia (SBPT) (c2024). Apenas 12,3% dos asmáticos brasileiros estão com a doença bem controlada.
